# Mitochondrial Ca^2+^ Signaling in Health, Disease and Therapy

**DOI:** 10.3390/cells10061317

**Published:** 2021-05-25

**Authors:** Lorenzo Modesti, Alberto Danese, Veronica Angela Maria Vitto, Daniela Ramaccini, Gianluca Aguiari, Roberta Gafà, Giovanni Lanza, Carlotta Giorgi, Paolo Pinton

**Affiliations:** 1 Laboratory for Technologies of Advanced Therapies (LTTA), Department of Medical Sciences, University of Ferrara, 44121 Ferrara, Italy; mdslnz@unife.it (L.M.); dnslrt@unife.it (A.D.); vttvnc@unife.it (V.A.M.V.); rmcdnl@unife.it (D.R.); grgclt@unife.it (C.G.); 2 Department of Neuroscience and Rehabilitation, University of Ferrara, 44121 Ferrara, Italy; gianluca.aguiari@unife.it; 3 Department of Translational Medicine, University of Ferrara, 44121 Ferrara, Italy; roberta.gafa@unife.it (R.G.); giovanni.lanza@unife.it (G.L.)

**Keywords:** mitochondria, Ca^2+^, cancer, cardiovascular diseases, neurodegenerative diseases, mPTP, therapy

## Abstract

The divalent cation calcium (Ca^2+^) is considered one of the main second messengers inside cells and acts as the most prominent signal in a plethora of biological processes. Its homeostasis is guaranteed by an intricate and complex system of channels, pumps, and exchangers. In this context, by regulating cellular Ca^2+^ levels, mitochondria control both the uptake and release of Ca^2+^. Therefore, at the mitochondrial level, Ca^2+^ plays a dual role, participating in both vital physiological processes (ATP production and regulation of mitochondrial metabolism) and pathophysiological processes (cell death, cancer progression and metastasis). Hence, it is not surprising that alterations in mitochondrial Ca^2+^ (mCa^2+^) pathways or mutations in Ca^2+^ transporters affect the activities and functions of the entire cell. Indeed, it is widely recognized that dysregulation of mCa^2+^ signaling leads to various pathological scenarios, including cancer, neurological defects and cardiovascular diseases (CVDs). This review summarizes the current knowledge on the regulation of mCa^2+^ homeostasis, the related mechanisms and the significance of this regulation in physiology and human diseases. We also highlight strategies aimed at remedying mCa^2+^ dysregulation as promising therapeutical approaches.

## 1. Introduction

Mitochondria are membrane-bound cellular organelles that are often referred to as the cell powerhouse. Indeed, they play a primary role in generating most of the chemical energy (ATP) that acts as fuel for the cell through oxidative phosphorylation. Undoubtedly, energy production represents only the very tip of the iceberg in terms of mitochondrial function. In fact, these highly dynamic structures integrate a wide spectrum of cellular activities, such as metabolism, muscle contraction, neurotransmitter release, antioxidant defense, cell signaling, autophagy and programmed cell death [1,2,3]. It is widely recognized that these organelles are not static and passive; rather, they constantly change their shape in response to environmental changes and stresses through fission and fusion processes [4]. Thus, they exert both vital and lethal functions in physiological and pathological scenarios. Mitochondria are considered efficient in decoding intracellular signals, of which Ca^2+^ is one of the most important [5]. In fact, they control and balance Ca^2+^ influx and efflux. Specifically, the mitochondrial calcium Ca^2+^ uniporter (MCU) complex (MCUC) ensures Ca^2+^ uptake, while the Na^+^/Ca^2+^ exchanger (NCLX) and H^+^/Ca^2+^ exchanger (HCX) supervise its extrusion [6,7]. Under resting conditions, the Ca^2+^ concentration inside mitochondria reaches levels comparable to those in the cytoplasm (100–200 nM). However, after stimulation with agents that increase Ca^2+^ levels, 10- to 20-fold more Ca^2+^ can accumulate in mitochondrial than in the cytosolic compartment. The presence of dynamic membrane contacts between mitochondria and the endoplasmic reticulum (ER; the main Ca^2+^ stores store inside the cells), termed mitochondrial-associated membranes (MAMs), and a highly Ca^2+^-selective channel located in the inner mitochondrial membrane (IMM) allow a large amount of Ca^2+^ to enter these organelles. Nonetheless, Ca^2+^ ions need to be rapidly extruded to restore the basal state. This process is guaranteed by a complex system of Ca^2+^ antiporters, represented by NCLX and HCX activity. Excessive calcium uptake or impairments in calcium efflux can produce deleterious effects on mitochondrial functionality [8]. In fact, excessive transfer of Ca^2+^ from the ER to mitochondria via inositol 1,4,5-trisphosphate (IP3) receptor channels (IP3Rs) leads to mCa^2+^ overload and subsequent mitochondrial permeability transition pore (mPTP) opening. Persistent opening of the mPTP provokes inner mitochondrial membrane (IMM) depolarization and matrix swelling, thus inducing outer mitochondrial membrane (OMM) rupture. Then, cytochrome c is released, inducing apoptotic cell death [9,10]. Conversely, decreased expression of MCU leads to a lower mCa^2+^ uptake thus causing a reduction of mPTP opening and preventing apoptotic factors release [11,12,13]. It is clear that alterations in or disruption of mCa^2+^ homeostasis could produce a pathological scenario. Indeed, mCa^2+^ dysfunction has been extensively implicated in various common diseases, including neurodegenerative diseases (such as Alzheimer’s disease (AD), Parkinson’s disease (PD) and Huntington’s disease (HD)), cardiovascular diseases (CVDs; ischemia/reperfusion (IR), injury (IRI), cardiac hypertrophy, cardiomyopathies and arrythmia) and, last but not least, cancer. Under pathological conditions in which mCa^2+^ overload triggers cell damage, as in IRI and neurological disorders, drugs that inhibit increases in mCa^2+^ levels might be beneficial. On the contrary, molecules that enhance mCa^2+^ overload could be useful in scenarios where reductions in mCa^2+^ levels allow cancer cells to evade apoptosis. Although mitochondria have a central role in human health and disease, successful therapies targeting these organelles are still not available.

## 2. Mitochondrial Calcium Homeostasis

### 2.1. Mitochondrial Ca^2+^ Influx

Mitochondria are characterized by two functional and distinct membrane systems, i.e., the OMM and the IMM, and folded cristae that enclose the mitochondrial matrix. mCa^2+^ homeostasis is tightly regulated by proteins localized in the IMM and OMM and by crosstalk with the ER [14]. This is essential for cell functions and is guaranteed by a dynamic equilibrium between mCa^2+^ influx and efflux [15]. Over the past years, this topic has been deeply reviewed, and interested readers are referred to recent reviews [15,16,17]. Briefly, Ca^2+^ diffusion across the OMM occurs via porin-like proteins named voltage-dependent anion channels (VDACs; the isoforms VDAC1-3) [18]. Then, Ca^2+^ enters the mitochondrial matrix via the MCUC, which is located in the IMM, thanks to a high electronegative potential (∼180 mV). The molecular identity of this channel was revealed only 10 years ago by Rizzuto’s and Mootha’s groups [19,20] after MICU1 was identified as a regulator rather than the channel itself [21]. The MCU gene, also known as CCDC109A, encodes a 40 kDa protein with two coiled-coil domains and two transmembrane domains separated by a short loop [22]. It is now widely accepted that MCU is the principal component of a larger macromolecular complex named MCUC. MCUC is composed of MCU; MCUb, an MCU paralog that acts as its negative regulator [23]; EMRE (essential MCU regulator), which is fundamental for the complex stabilization [24]; and the associated regulators MICU1, MICU2 and MICU3. MICU1 functions as a gatekeeper for the MCU complex, stabilizing the MCU complex in the closed state and thus setting the threshold for mCa^2+^ uptake [25,26]. MICU2 and MICU3 are two MICU1 paralogs. While MICU2 is localized at the mitochondrial intermembrane space (IMS) and is widely expressed in most mammalian tissues as MICU1, MICU3 mitochondrial localization prediction has a lower confidence [27,28] and is prevalently expressed in the nervous system and skeletal muscle [27,29]. MICU2 forms heterodimers with MICU1, which is responsible for the sigmoidal response to increasing cytosolic Ca^2+^ (cytCa^2+^) concentration [26,30]. Regarding MICU3 function, Patron and colleagues recently demonstrated that it forms a disulfide bond-mediated dimer with MICU1 but not with MICU2, acting as a strong MCU stimulator without gatekeeping function [31]. The affinity of MCUC for Ca^2+^ is very low (*K*_D_ of 20–30 μM under physiological conditions). Thus, for a significant mCa^2+^ influx, cytCa^2+^ levels should be 5–10 μM, but these values have never been detected in live cells. This conundrum was explained by the presence of MAMs, where mitochondria are in close contact with the ER [7]. At MAMs level, the release of Ca^2+^ content from the ER produces microdomains of high [Ca^2+^] which allow a rapid accumulation of Ca^2+^ inside mitochondria [32]. 

### 2.2. Mitochondrial Ca^2+^ Efflux

The existence of pathways for the extrusion of Ca^2+^ from mitochondria was revealed in the 1970s [33,34]. Ca^2+^ efflux from the mitochondrial matrix depends on two mechanisms. One involves a ubiquitous HCX [33], and the other involves NCLX [34], which is mostly expressed in excitable tissues (muscle and brain). The molecular identity of HCX is still debated, but recent works have proposed that LETM1 functions as the mitochondrial electroneutral H^+^/Ca^2+^ antiporter [17,35]. However, this finding is not universally accepted [36,37]. In 2010, Palty et al. found that the SLC8B1 gene encodes an IMM-localized protein that is responsible for both Li^+^- and Na^+^-dependent Ca^2+^ clearance from the mitochondrial matrix and is thus named NCLX [38]. To date, the role of NCLX has clearly been proven in different in vitro cellular models. Notwithstanding this evidence, to date, animal models in which NCLX is absent are unavailable. Thus, future evidence will be crucial to better analyze and elucidate how mCa^2+^ homeostasis is achieved under pathophysiological conditions [15]. Recent evidence suggests that HCX and NCLX are not the only two molecules responsible for Ca^2+^ efflux. As stated above, prolonged mPTP opening might lead to cell death. Despite this finding, it has been reported that in certain circumstances, transient mPTP opening can aid Ca^2+^ extrusion [39,40], although this hypothesis is not widely accepted [41].

### 2.3. Physiological Role of Mitochondrial Ca^2+^

**Figure 1:**
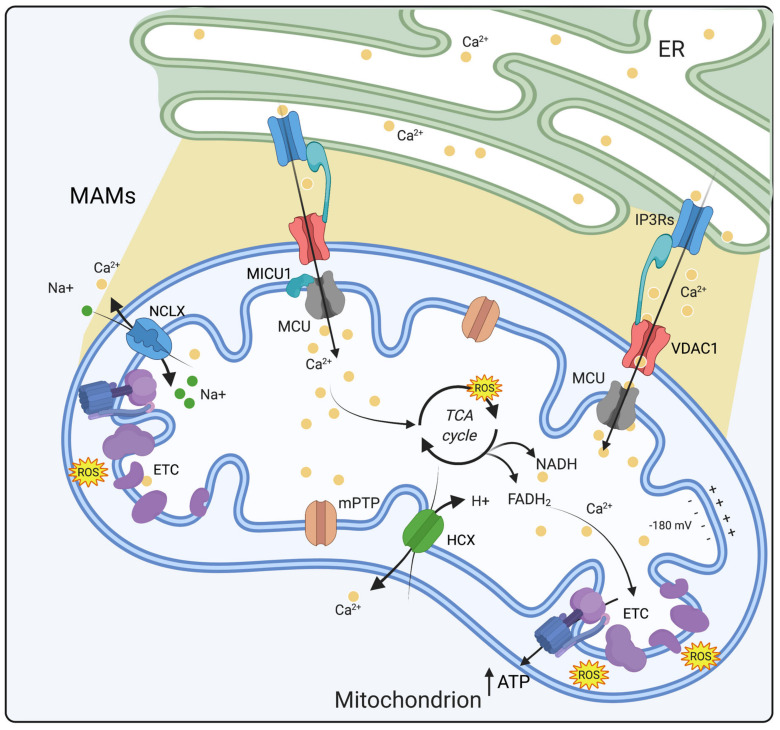
Mitochondrial calcium homeostasis. mCa^2+^ homeostasis is tightly regulated by influx and efflux mechanisms. Ca^2+^ enters into the mitochondrial matrix via MCU and through a high electronegative potential (−180 mV) while its extrusion depends on NCLX and HCX exchangers. Within the matrix, Ca^2+^ stimulates the activity of three dehydrogenases of the Krebs cycle and ATP production. Ca^2+^ ions are depicted as yellow dots. Abbreviations: ER, endoplasmic reticulum; MAMs, mitochondria associated membranes; ETC, electron transport chain; MCU, mitochondrial calcium uniporter; VDAC1, voltage-dependent anion channel 1; ATP, adenosine triphosphate; MICU1, mitochondrial calcium uptake 1; IP3Rs, inositol-1,4,5-trisphosphate receptors; ROS, reactive oxygen species; mPTP, mitochondrial permeability transition pore; NCLX, Na^+^/Ca^2+^ exchanger; HCX, H^+^/Ca^2+^ exchanger (Created with Biorender.com).

As mentioned above, mCa^2+^ homeostasis is tightly regulated by influx and efflux mechanisms and it affects oxidative metabolism, generation of mitochondrial ROS and mPTP opening. The accumulation of Ca^2+^ within mitochondria stimulates important functions of the organelle, including ATP production through oxidative phosphorylation. Indeed, mCa^2+^ regulates the tricarboxylic acid (TCA) cycle by modulating the activity of three key enzymes of mitochondrial metabolism: ketoglutarate dehydrogenase (KGDH), isocitrate dehydrogenase (IDH) and pyruvate dehydrogenase (PDH). This effect boosts the synthesis of NADH and FADH_2_, leading to an enhanced respiratory chain activity and thus a subsequent increase in H^+^ pumping. The electrochemical energy produced is then used to drive ATP synthesis by complex V (ATP synthase) (Figure 1) [42,43]. Under both physiological and pathological conditions, the mitochondrial electron transport chain (ETC), especially complex I and III, has primary responsibility for ROS production. Even though mitochondrial ROS (mROS) have been mainly considered as detrimental by-products of oxidative metabolism, they are now recognized as important signaling molecules, (at subtoxic levels) regulating several cellular activities [44]. mCa^2+^ uptake by increasing the metabolic rate and ETC activity drives ROS production [45]. This signaling axis works efficiently within a physiological window of [Ca^2+^]. As a result, when [Ca^2+^] exceeds this threshold, mROS production becomes harmful and deleterious for mitochondrial bioenergetics and cell functions. ROS formation may be promoted by mCa^2+^ either directly by stimulating mROS-generating enzymes such as glycerol phosphate and KGDH or indirectly as in the case of nitric oxide synthase (NOS) activation which inhibits complex IV, leading to excessive mROS generation [46]. Hence, the strict cooperation between mCa^2+^ and mROS signaling seems to have important implications for maintaining cellular homeostasis [43]. Ca^2+^ signaling plays an essential role in excitable cells as it controls cardiac and skeletal muscle contraction, and synaptic transmission (reviewed in [47]). In view of the fact that neurons regulate extremely important functions such as the transmission of depolarized signals, synaptic plasticity and metabolism, they require a precise spatiotemporal control of Ca^2+^ [48]. Ca^2+^ influx into neurons occurs principally through ligand-gated glutamate receptors such as N-methyl-d-aspartate receptors (NMDAR) and voltage-dependent ion channels (VDCCs), as well as the release of Ca^2+^ from intracellular stores [49]. Remarkably, neurons almost exclusively rely on mitochondrial oxidative phosphorylation (OXPHOS) as the main source of ATP production, and mCa^2+^ uptake guarantees activity-dependent regulation of cellular energy metabolism [50]. Considering that neurons are particularly sensitive to [Ca^2+^] oscillations, even small variations in Ca^2+^ homeostasis can produce deleterious consequences, leading to alterations in physiological neuronal activity such as in aging [51] and neurodegeneration [48]. In the heart, most of the energy needed for cardiac cells excitation and contraction is produced within mitochondria through OXPHOS which, as mentioned above, is a Ca^2+^-modulated process [16]. The strategic positioning and presence of mitochondria in cardiac cells (over 30% of the cardiac mass) [52] ensure an efficient ATP production to support contractility, metabolism and ion homeostasis [53]. A detailed explanation about the role of mitochondria in the physiology of cardiac cells appears in Section 3.3.

## 3. Mitochondrial Calcium Dyshomeostasis

### 3.1. Dysregulation of Mitochondrial Ca^2+^ Signaling in Cancer and the Cell Cycle

**Figure 2:**
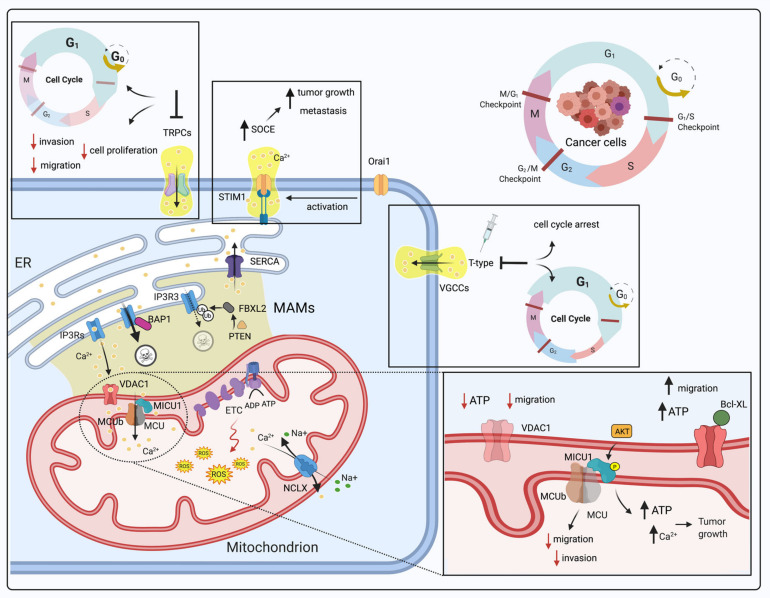
Schematic view of the role of Ca^2+^ dysregulation in cancer and the cell cycle with a particular focus on alterations in mCa^2+^ levels. Ca^2+^-regulating proteins and channels are involved in cell cycle progression, and their dysregulation leads to alterations in the cell cycle. STIM1/ORAI1-mediated augmentation of SOCE promotes tumor growth and metastasis; VGCCs are involved in cell proliferation regulation, and T-type channel inhibition provokes cell cycle arrest followed by a significant increase in the number of cells in G1 phase and a decrease in the number of cells in S phase; TRPC1 inhibition reduces the adhesion, invasion and proliferation of different cancer cell lines and G(0)/G(1) cell cycle arrest of glioma and lung carcinoma cell lines. MCU silencing inhibits cell migration and invasion without affecting proliferation rates or apoptosis levels. MCUb silencing limits proliferation, migration, and invasion, as well as glioma progression in vivo. Loss of VDAC1 leads to ATP depletion, which results in decreased cell growth and migration in several cancer cell lines both in vitro and in vivo. The interaction between VDAC and Bcl-XL is responsible for increased ATP production in breast cancer cells, which leads to an increased migration rate. AKT-mediated phosphorylation of MICU1 causes an increase in mCa^2+^ content under basal conditions and ROS production, which promotes AKT-mediated tumor growth. At MAMs, IP3R3 FBXL2-dependent degradation is enhanced in cancer cells upon loss of PTEN, resulting in apoptosis resistance. The tumor suppressor BAP1 is capable of binding, deubiquitylating and stabilizing IP3R3 channels, modulating Ca^2+^ release into the cytosol and then into mitochondria and thus promoting cell death. Abbreviations: SOCE, store-operated calcium entry; VGCCs, voltage-gated calcium channels; TRPC1, transient receptor potential channel 1; MCU, mitochondrial calcium uniporter, MCUb, mitochondrial calcium uniporter b subunit; VDAC1, voltage-dependent anion channel 1; ATP, adenosine triphosphate; Bcl-XL, B-cell lymphoma XL; AKT, protein kinase B; MICU1, mitochondrial calcium uptake 1; IP3R3, type 3 inositol-1,4,5-trisphosphate receptor; FBXL2, F-box and leucine-rich repeat protein 2; BAP1, BRCA1-associated protein 1; PTEN, phosphatase and tensin homolog; ROS, Reactive oxygen species (Created with Biorender.com).

Cell death is necessary for life. This, which might seem to be contradictory, is the key to understanding how the cell makes delicate prolife and prodeath decisions to preserve the health of the organism. When a cell is no longer needed, a plethora of cellular signaling pathways activate a program that ultimately leads to self-destruction, giving cell death a connotation that is anything but negative. Ca^2+^ signaling is undoubtedly one of the most important mechanisms involved in these decisions, and especially in recent years, it has been shown that the dysregulation of Ca^2+^ homeostasis may result in tumor pathologies [54]. Because they intervene in important process in cancer progression, such as proliferation and invasiveness [55], an increasing number of Ca^2+^-regulating proteins are being identified as oncogenes and tumor suppressors [56]. Therefore, it is not surprising that many of the previously mentioned Ca^2+^-related proteins and channels are involved in cell cycle progression and that their dysregulation leads to aberrant cell cycle activity (Figure 2) [55]. Duplication of genetic material and cell division in mammals is guaranteed by the cell cycle, a highly organized and regulated process that can be divided into four distinct phases (G0/G1, S, G2 and M) and controlled by several cyclin-dependent kinases (CDKs) that act in complex with their cyclin partners [57]. Ca^2+^ ions have been shown to affect the activity of several CDK and CDK–cyclin complexes; for example, Ca^2+^ and calmodulin (CaM) exert effects on the regulation of the expression of CDK1, CDK2 and cyclin B in human T lymphocytes [58]. The quiescent phase (G0) is a state of cell cycle arrest; in most adult tissues, cells can be either in transient or permanent G0 phase. CDK4 and CDK6 trigger quiescent cells to re-enter the cell cycle in S phase, in which DNA replication occurs. Ca^2+^/calmodulin-dependent protein kinase (CaMKI), through CaM, is implicated in the regulation of the cyclin D1-CDK4 complex in fibroblasts [59], which in turn regulates retinoblastoma protein (RB1), the main inhibitor of DNA synthesis [60]. The cyclin D-CDK4/6 complex is hyperactivated in many types of human cancers in which the CDK4/6–RB pathway is dysregulated [61].

Alterations in the intracellular Ca^2+^ concentration have biphasic effects: on the one hand, an increase in the cytCa^2+^ level promotes cell migration and is an important feature of cancer cells’ metastatic behavior; on the other hand, reduced Ca^2+^ transfer via MAMs and a decrease in store-operated calcium entry (SOCE) modulate cell death by contributing to acquired resistance to apoptosis of primary tumors [62].

Since the identification of the key molecules involved in SOCE, there has been extreme interest in determining the role of this Ca^2+^ influx pathway in tumor onset and progression. A clear example is represented by the STIM1-ORAI1 Ca^2+^ flux pathway that under physiological conditions promotes the G1 to S transition and inhibits the S to G2 transition [63]. Although STIM/ORAI1-mediated augmented SOCE has been reported to promote tumor growth and metastasis in many cancer types, STIM1 drives growth arrest in human rhabdomyosarcoma and rhabdoid tumor cell lines [64], ORAI1 facilitates apoptosis of PCa cells and the knockdown of ORAI1 leads to drug resistance [65].

Karacicek et al. reported that STIM1 overexpression facilitates cancer cell survival also by preventing mCa^2+^-dependent enzymatic activity in which MCU requires much higher cytCa^2+^ concentration [66].

Low voltage-activated T-type channels are members of the voltage-gated calcium channel (VGCC) family, a group of voltage-gated ion channels that open their calcium-selective channel pores as a result of membrane potential depolarization, allowing Ca^2+^ influx into the cell. It has been shown that cytCa^2+^ elevations were paralleled by mitochondrial calcium elevations which were also increased by T-type calcium channels overexpression [67]. VGCCs are associated with cell proliferation regulation [68], and it has been shown that low voltage-activated T-type channel inhibitors provoke cell cycle arrest accompanied by a significant increase in the number of G1 phase cells and a decrease in the number of S phase cells in human melanoma cells [69]. Mibefradil, a pharmacological inhibitor of T-type channels, was shown to have a similar cell cycle arrest effect to increase G0/G1 phase distribution in a colon cancer model [70] and in two ovarian cancer cell lines [71].

Transient receptor potential (TRP) channels form a versatile family of ion channels, the majority of which are Ca^2+^-permeable, playing a significant role in the cell cycle. TRP channels exert their effects by regulating gene transcription and shaping other cellular processes, such as proliferation, cell motility and apoptosis [72]. For instance, TRPC1 is involved in various tumor pathologies in a cancer stage-specific manner; inhibition of the expression or activity of TRPC1 mitigates cell adhesion and invasion ability in nasopharyngeal carcinoma [73], inhibits the migration of HCT-116 colon cancer cells and the proliferation of MDA-MB-468 breast cancer cell lines and leads to G(0)/G(1) cell cycle arrest of glioma and lung carcinoma cell lines [74]. Interestingly, resiniferatoxin, a TRPV1 agonist, has been shown to promote the inhibition of mitochondrial function and induction of apoptosis in pancreatic cancer cells [75].

As we have described in the introductory section of this work, mitochondria and the MAMs compartment have been found to be very important for Ca^2+^ signaling, especially in recent years. Among the several pathologies that can arise from perturbations in Ca^2+^ homeostasis at this level, tumors are being studied in depth. Mitochondria are major sites of ROS generation, which occurs largely at complexes I and III of the ETC. An increase in ROS production often arises when electron transport function is compromised, leading to excessive leakage of electrons which then react with oxygen to form superoxide [76].

It is well known that mROS participate in stress signaling under physiological conditions and contribute to the induction of nuclear and/or mitochondrial DNA mutations that stimulate neoplastic transformation. Indeed, mitochondrial ROS strengthen the tumorigenic phenotype and trigger additional mutation accumulation, leading to metastatic behavior [77]. The tumor suppressor protein p53 is a mitochondrial ROS production modulator; however, it is not clear whether its ability to regulate mitochondrial ROS production leads to cell death or stimulates malignancy [78,79]. The antitumoral potential of p53 is well demonstrated by its ability to induce G1 and postmitotic cell cycle arrest and apoptosis [80].

ERK1/2, a kinase belonging to the MAPK family, is activated through a sequential phosphorylation cascade that leads to the signal amplification and the transduction of signals to mitochondria [81]. ERK1/2 acts on FOXO transcription factors that trigger the expression of multiple target genes involved in tumor suppression to induce apoptosis [82] and cell cycle regulation involving p27kip1 and cyclin D [83]. The expression of FOXO3a is linked to tumor progression suppression, while inhibition of its expression promotes tumor progression, angiogenesis and cell transformation [84].

The role of the channel MCU in tumors is multifaceted and strongly debated [85]. In 2013, Marchi et al. found that miR-25, an MCU-targeting microRNA, is overexpressed in colon cancer, where its overexpression correlates with a decrease in Ca^2+^ uptake and promotes cancer cell survival by enhancing proliferation [86]. However, in vitro studies have shown that MCU silencing in HeLa and Hs578T breast cancer, triple-negative breast cancer and hepatocellular carcinoma (HCC) cells drastically inhibits cell migration, motility and invasion without affecting basal proliferation rates or apoptosis levels [87,88,89]. In MCU-deficient cells, cell cycle progression is delayed at the G1-S phase transition, a stage in which mitochondrial fusion and increased mCa^2+^ uptake occur under physiological conditions [90].

With reference to MCU complex subunits, a pool of activated AKT can localize at the IMS, where it phosphorylates MICU1. Phosphorylation of MICU1 abolishes its gatekeeping function, leading to higher mCa^2+^ content under basal conditions and ROS production and thus to AKT-mediated tumor growth (Table 1) [91]. The expression levels of MCUb are inversely associated with overall survival in glioma. Interestingly, it has been reported that MCUb silencing limits glioma cell proliferation, migration, and invasion, as well as glioma progression in vivo [92].

As previously mentioned, VDAC is a crucial protagonist of mCa^2+^ homeostasis, facilitating the flow of Ca^2+^ into and out of mitochondria [93]. Loss of the VDAC isoform VDAC1 leads to ATP depletion, which results in decreased cell growth and migration in colon, lung and pancreatic cancer cells both in vitro and in vivo [94]. The interaction between VDAC and the antiapoptotic protein Bcl-XL is responsible for breast cancer cell-induced increases of ATP production, the main promoter of migration [95].

Some essential proteins have also been demonstrated to affect Ca^2+^ flux into mitochondria in an ER-mediated manner, and among these proteins, IP3Rs undoubtedly play a leading role. Even if Ca^2+^ overload triggers the mitochondrial apoptotic pathway, a degradation process named autophagy is activated as a result of poor mCa^2+^ uptake caused by insufficient Ca^2+^ transfer from the ER [96].

Phosphatase and tensin homolog (PTEN), a MAM-localized tumor suppressor, enhances Ca^2+^ release from the ER and can compete with FBXL2, an E3-ubiquitin ligase F-box protein, to bind to IP3R3 to prevent its degradation.

Our group has demonstrated that IP3R3 FBXL2-dependent degradation is enhanced in cancer cells with poor PTEN expression, thus resulting in the inhibition of apoptosis [97].

Moreover, BRCA1-associated protein-1 (BAP1) is another protein with tumor suppressive properties that has been demonstrated to bind, deubiquitylate and stabilize the activity of the IP3R3 channel in the ER, modulating Ca^2+^ release into the cytosol and then into mitochondria and thus promoting apoptosis (Table 1 and Figure 2) [98].

Especially in recent years, it has become evident that calcium signaling, in particular the mitochondrial proteins and pathways involved in it, is a good target for the development of effective and targeted anticancer therapies. Since many mCa^2+^ channels/pumps/transporters play a role in normal physiological processes and cell cycle progression, one challenge for drug development is the design of drugs that regulate the cell cycle progression of malignant cells.

### 3.2. Dysregulation of Mitochondrial Ca^2+^ Signaling in Neurodegenerative Diseases

Neurodegenerative diseases are a group of heterogeneous disorders characterized by the progressive and selective death of neuronal subtypes. A growing body of evidence suggests that alterations in Ca^2+^ homeostasis, in particular the dysregulation of mCa^2+^ signaling, are implicated in different neurodegenerative diseases such as AD, PD and HD [99,100,101]. Neuronal Ca^2+^ regulation is extremely important even before the appearance of the pathological characteristics of these diseases [99,102,103].

**Figure 3:**
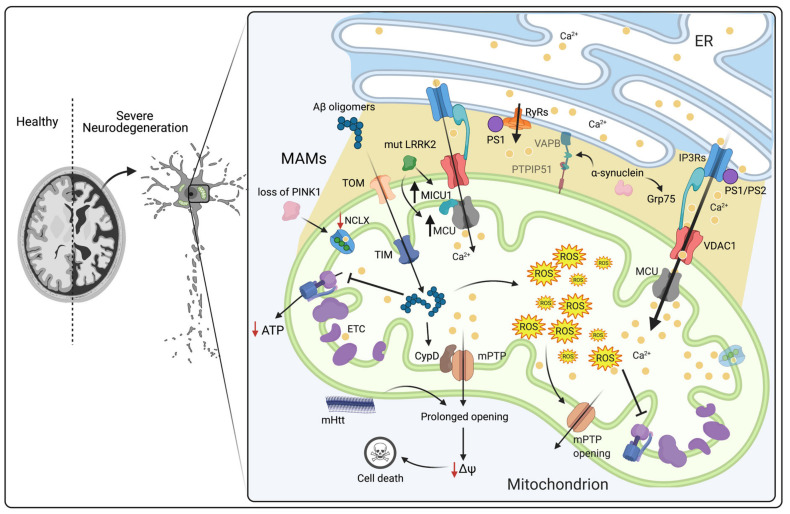
Schematic view of the role of mCa^2+^ dysregulation in neurodegenerative diseases. In AD, Aβ oligomers enter mitochondria via the translocases TOM and TIM. Once inside the matrix, they interact with specific intramitochondrial targets: (i) respiratory chain complexes III and IV, leading to ATP synthesis reduction, and (ii) CypD, leading to mPTP opening, ΔΨ_m_ collapse and activation of cell death. Excessive levels of mCa^2+^ can enhance ROS production, reduce the ΔΨm, and stimulate mPTP opening, thus leading to the release of proapoptotic factors. In HD, mHtt decreases the Ca^2+^ threshold necessary to trigger mPTP opening, preventing the binding of mPTP inhibitors and consequently augmenting its activation by increasing the binding affinity of CypD and Ca^2+^. In PD, α-synuclein interacts with the chaperone Grp75, thus contributing to enhancement of ER–mitochondria communication. Overexpression of wt and mutant α-synuclein leads to the destruction of VAPB-PTPIP51 tethers through its bond with VAPB, causing a decrease in the ER–mitochondria association. PINK1 deficiency results in mitochondrial calcium overload and subsequent ROS production due to negative regulation of NCLX. Mutant LRRK2 induces transcriptional upregulation of MCU and MICU1, thus leading to mCa^2+^ accumulation. Mutant PS1 and PS2 interact and modulate the IP3R Ca^2+^ release channel causing a strong stimulatory effect on its gating activity. PS1 N-terminal region can interact with ryanodine receptor (RyR) and enhance its activity. Abbreviations: ER, endoplasmic reticulum; MAMs, mitochondria-associated membranes; mPTP, mitochondrial permeability transition pore; CypD, cyclophilin D; ROS, reactive oxygen species; TIM, translocase of the inner membrane; TOM, translocase of the outer membrane; MICU1, mitochondrial calcium uptake 1; MCU, mitochondrial calcium uptake; NCLX, Na^+^/Ca^2+^ exchanger; Grp75, glucose-regulated protein 75; IP3R3, inositol-1,4,5-trisphosphate receptor type 3; ETC, electron transport chain; PINK1, PTEN-induced kinase 1; LRRK2, leucine-rich repeat kinase 2; mHtt, mutant Htt; ΔΨ_m_, mitochondrial membrane potential; PS1, presenilin 1; PS2, presenilin 2 (Created with Biorender.com).

Alzheimer disease is a multifactorial and chronic neurodegenerative disease that is characterized by the loss of cognitive functions and memory. There are two distinct forms of AD: hereditary forms (∼10%) characterized by an early onset, known as familial AD (FAD), and sporadic AD (∼90%) (SAD) which usually develops beyond the age of 60 [104]. SAD is characterized by alterations in several genes. Among these, the apolipoprotein E (APOE) gene that produces the ε4 allele of the APOE (APOE4 variant) is one of the most studied [105]. APOE4 expression can promote an increase in ER–mitochondria contact sites, causing an elevation of mCa^2+^ and cytCa^2+^ levels [104]. FAD is characterized by mutations in amyloid precursor protein (APP), Presenilin-1 (PS1) and Presenilin-2 (PS2). The most influential hypothesis for AD is based on the abnormal proteolytic cleavage of APP, which induces the formation and accumulation of amyloid β-peptide (Aβ), leading to the development of extracellular plaques [106,107]. In addition, intracellular neurofibrillary tangles are formed through the aggregation of the microtubule-associated protein tau [108]. These events ultimately lead to synaptic dysfunction and progressive neuronal death in brain regions dedicated to learning processes and memory [109,110,111]. Interestingly, Aβ neurotoxicity has also been associated with disruption of intracellular Ca^2+^ signaling [112]. Indeed, several studies have shown that amyloidogenic pathway induces alterations of the mechanisms involved in memory, learning and intraneuronal Ca^2+^ signaling [113,114]. As a matter of fact, in the early 1990s, it was shown that enhanced InsP3-mediated Ca^2+^ signaling was a prognostic diagnostic feature in AD-derived cells [115]. Subsequently, Cheung et al. have shown that mutant PS1 and PS2 interact and modulate the IP3R Ca^2+^ release channel. This interaction has a strong stimulatory effect on its gating activity that ultimately leads to an abnormal Ca^2+^ signaling response to agonist stimulation (Table 1) [116]. Similarly, mutant PS1 N-terminal region can interact with ryanodine receptor (RyR) and enhance its activity both in vitro and in animal models of AD (Figure 3) [117,118]. In fact, studies carried out on young neurons of 3xTg-AD mice revealed a selective increase of about fivefold of the RyR2 isoform in relation to control non-transgenic mice, influencing the plasticity and synaptic activity in AD mouse models [117]. All these alterations that affect ER-Ca^2+^ release have an indirect effect on mCa^2+^ uptake [119]. Therefore, the enhanced transfer of Ca^2+^ between ER and mitochondria results in an increase in mCa^2+^. The subsequent mCa^2+^ overload, as already mentioned above, triggers mPTP opening and the release of proapototic factors [120,121], thus contributing to neurotoxicity [112]. In addition, early onset of the disease is caused by Aβ oligomers acting directly on the increase in mCa^2+^ uptake [119]. Aβ oligomers can be transported into mitochondria via translocase of the outer membrane (TOM) and translocase of the inner membrane (TIM). Once inside the mitochondria, they interact with specific intramitochondrial targets, leading to a reduction in respiratory chain complex III and IV activity [122,123,124,125]. In vivo studies have shown that mCa^2+^ overload is elicited by a direct action of Aβ oligomers that triggers apoptosis through ATP synthesis inhibition, mPTP opening and ΔΨm collapse [126,127,128] (Figure 3). Notably, several studies have demonstrated that in AD patients’ brains and in transgenic mouse models of AD, neuronal injuries and the decline in cognitive functions are caused by the interaction of mitochondrial Aβ with cyclophilin D (CypD). CypD is a prolyl isomerase situated in the mitochondrial matrix and is an integral component of the mPTP. Its interaction with Aβ stimulates the opening of the mPTP and thus promotes cell death. Therefore, CypD deficiency protects neurons from oxidative stress, Aβ-induced cell death, synaptic dysfunction and deficits in memory and learning [129]. On the one hand, Calvo-Rodriguez M. et al. have demonstrated in healthy brain that the Aβ soluble oligomers induces mCa^2+^ increase through MCU. Given that, excessive Ca^2+^ taken up by mitochondria may lead to the opening of the mPTP and eventually to neuronal cell death. Conversely, the inhibition of MCU due by Ru360, prevents mCa^2+^ overload. Hence, proposing mPTP inactivation and the inhibition of MCU as a new potential therapeutic approach for AD [119]. On the other hand, Jadiya P. and colleagues have proposed another mechanism for Aβ mediated Ca^2+^ overload. Specifically, they have shown that neuronal deletion of the mitochondrial NCLX induces an increase in amyloidosis and tau pathology, accelerating memory degeneration. In 3xTg-AD triple mutant mice (which harbor mutations in PS-1, APP and tau) and in the brains of human patients with AD, the levels of NCLX are reduced, leading to an increase in mCa^2+^ concentration (Table 1). It is interesting to note that through recovery of NCLX expression, it is possible to reduce mCa^2+^ overload and consequently to prevent cognitive decline and AD-related pathology [130]. Additionally, in AD models, adverse effects have been observed in mitochondrial bioenergetics induced by a reduction of mCa^2+^ signal. In FAD, the reduction of mCa^2+^ is mediated by PS2 mutations which decrease the ER Ca^2+^ content [131,132], thus leading to an increase in neuronal sensitivity to excitotoxicity and neuronal bioenergetics [124,132]. Interestingly, PS1 and PS2 are not distributed homogeneously in the ER, but are highly enriched in MAMs [133,134]. Given this, MAM function and ER–mitochondrial connectivity are up-regulated in AD. Pera et al. have demonstrated that y-secretase is enriched at MAMs and is responsible for cutting the C-terminal fragment of APP (C99) that generates Aβ [135,136]. Consequently, in cells of AD patients, high levels of C99 have been detected in MAMs fraction, besides alterations in MAMs function and structure [135]. Based on this, in AD, the C99 accumulation in MAMs is upstream of mitochondrial dysfunction, suggesting an early role of mitochondrial dysfunction in the disease [137]. All these evidences suggest that altered ER–mitochondrial communication play a critical role in AD pathogenesis. In particular, mitochondrial function and mCa^2+^ are essential for neuronal function and survival.

In addition, HD manifests as psychiatric, dementia, behavioral, motor and cognitive abnormalities. It belongs to the family of N-terminal polyglutamine (polyQ) diseases [138,139,140]. This neurodegenerative disease is caused by alterations in huntingtin (Htt), a cytosolic protein that is expressed in various tissues [141]. Although the mechanisms underlying the onset of the disease remain unclear, several studies have shown that mitochondrial dysfunction plays a key role in HD pathogenesis [123,142,143,144,145,146]. In fact, it has been verified that in clonal striatal cell lines from wild-type and mutant homozygote knockin mice, the Htt protein is localized in the OMM. A similar observation was made in a human neuroblastoma cell line, suggesting that this is a common characteristic of different cell types and that mutant huntingtin (mHtt) may have a direct deleterious effect on mitochondria [147]. Indeed, N-terminal mHtt leads to a drastic reduction in Ca^2+^ concentration, which is required for the activation of mPTP and the release of cytochrome c. This suggests that mHtt significantly decreases the Ca^2+^ threshold necessary to trigger mPTP opening, preventing the binding of mPTP inhibitors and consequently improving mPTP activation by increasing the binding affinity of CypD and Ca^2+^ [148]. Panov et al. observed a deficit in mCa^2+^ buffering in mitochondria isolated from HD patients: these mitochondria showed a lower ΔΨ_m_ and depolarized at lower Ca^2+^ loads [149]. In contrast, other groups have demonstrated that Ca^2+^ uptake capacity is increased in brain-derived mitochondria and cultured neurons derived from the YAC128 HD mouse model [150,151]. In HD, stimulation of glutamate receptors leads to an increase in cytCa^2+^ levels in medium spiny neurons (MSNs). Through the MCUC, excessive cytCa^2+^ enters mitochondria, causing mPTP opening and hence apoptosis or mitochondrial DNA damage. Before the onset of neurodegeneration, in both HD mouse models and in HD patients, mitochondrial dysfunction and abnormal levels of mCa^2+^ and cytCa^2+^ have also been observed [152]. In conclusion, HD is associated with an early Ca^2+^ handling defect which plays a critical role in the pathogenesis of the disease. Particularly, the alterations of mCa^2+^ homeostasis have a crucial impact on selective neurons degeneration. However, the exact mechanisms involved in the pathogenesis of HD have not yet been fully clarified. PD, another common neurodegenerative disorder, affects 6.3 million people above the age of 60 [153]. This disease belongs to a group of neurodegenerative diseases known as synucleinopathies, which are characterized by the aggregation of α-synuclein (a small lipid-binding protein) into Lewy bodies. At the cellular level, it is characterized by a loss of dopaminergic neurons in the substantia nigra pars compacta (SNc). Several studies have confirmed that abnormalities in mCa^2+^ levels are linked to its pathogenesis (reviewed in [154,155,156,157]). However, in the past 10 years, more than 30 genes responsible for PD pathogenesis, such as α-synuclein, Parkin, PTEN-induced kinase 1 (PINK1) and leucine-rich repeat kinase 2 (LRRK2) [158,159], have been identified [160,161,162,163,164,165,166]. α-Synuclein, which is normally located in the cytosol and in mitochondria, is involved in physiological and/or pathological mitochondrial function [167]. Recent studies have illustrated that wild-type α-synuclein is also located at MAMs, where it interacts with the chaperone Grp75, thus contributing to ER–mitochondria communication [168,169,170]. Interestingly, Guardia-Laguarta and colleagues demonstrated that α-synuclein mutations can lead to an increase in mitochondrial fragmentation and a reduction in ER–mitochondria contact sites [168]. Conversely, Calì et al. showed that in SH-SY5Y and HeLa cells, α-synuclein positively affects Ca^2+^ transfer from the ER to mitochondria [170,171]. Nevertheless, overexpression of wt and mutant α-synuclein leads to the destruction of VAPB-PTPIP51 tethers through binding with VAPB, which causes a decrease in ER–mitochondria associations. This disruption lessens mitochondrial ATP production and interrupts Ca^2+^ exchange between ER and mitochondria [172]. Although the mechanism of action remains unclear, mutations in or variants of many genes increase susceptibility to PD. For instance, PINK1 and parkin are two PD-associated proteins that influence mitochondrial pathways of Ca^2+^ influx [173]. In dopaminergic neurons expressing mutant PINK1, mCa^2+^ levels are elevated and the number of ER–mitochondria contact sites is increased, leading to a progressive loss of neurons [174]. Physiologically, PINK1 regulates Ca^2+^ efflux from mitochondria via NCLX, while Parkin stimulates VDAC1. PINK1 is a mitochondrial serine/threonine protein kinase and is required for Parkin recruitment and stress-induced mitophagy [175,176]. However, mitochondria isolated from the brains of mice lacking PINK1 seem to be more vulnerable to cell death [177]. In addition, Gandhi et al. proved that PINK1 deficiency results in mitochondrial calcium overload and subsequent ROS production due to negative regulation of NCLX. Indeed, given the reduced mCa^2+^ capacity and increased levels of ROS, the threshold of mPTP opening is low, making neurons vulnerable to programmed cell death [178]. Parkin is an E3 ubiquitin ligase that plays a key role in mitophagy, a mechanism that selectively removes damaged mitochondria [179]. It has been shown that Parkin-deficient cells and fibroblasts expressing mutant Parkin from PD patients display reduced ER–mitochondria tethering, resulting in diminished mCa^2+^ uptake [180]. In contrast, Gautier et al. reported that the number of ER–mitochondria contacts is increased in primary fibroblasts from PARK2 knockout mice and PD patients with PARK2 mutations [181]. Mutations in LRRK2 contribute to development of PD. Indeed, several studies have shown that cortical neurons expressing mutant LRRK2 exhibit a major increase in excitatory neurotransmission, which occurs before dendritic shortening. Interestingly, patient fibroblasts expressing mutant LRRK2 show higher levels of MCU and MICU1 and increased depolarization-induced mCa^2+^ uptake (Figure 3). In fact, in fibroblasts from both PD and Parkinson’s disease dementia (PDD) patients, the expression of mutant LRRK2 induces the transcriptional upregulation of MCU and MICU1 but not MICU2 and NCLX (Table 1). Hence, strategies that target either MCU or NCLX may serve to normalize activity-dependent mitochondrial calcium flux to protect against neurodegeneration [182]. All these data underline that the disruption of MAMs and alterations in mCa^2+^ levels may contribute to the onset of PD.

### 3.3. Dysregulation of Mitochondrial Ca^2+^ Signaling in CVDs

CVDs are considered the leading cause of mortality in Western countries [183]. Multiple and complex factors are involved in the onset and development of cardiac disorders; however, in recent years, mitochondrial dysfunction has been recognized as the hallmark of heart physiopathology [184,185]. As previously stated, mitochondria are responsible for long-term Ca^2+^-buffering. Specifically, in the heart, mCa^2+^ flux plays an important role not only in myocardial energy production and mitochondrial metabolism by activating Ca^2+^-sensitive dehydrogenases (PDH, IDH, KGDH) [186,187], but also in the regulation of cardiomyocyte contractility [188,189]. Therefore, disturbances in mCa^2+^ homeostasis (increased or decreased levels) contribute to the onset and development of many CVDs, such as IR, cardiac hypertrophy, cardiomyopathies and arrythmia [185,190].

**Figure 4:**
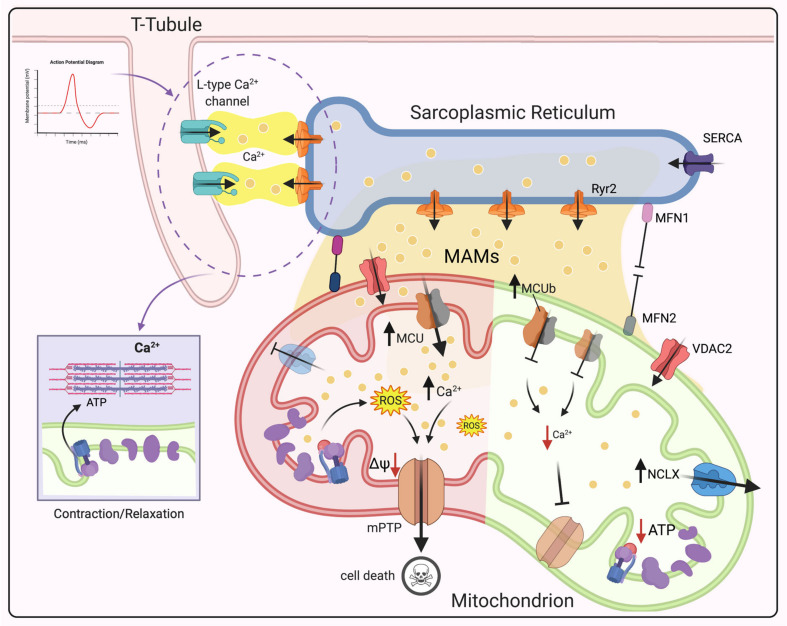
Schematic of the role of mCa^2+^ dysregulation in CVDs. Under physiological conditions, after sarcolemma depolarization, the opening of L-type Ca^2+^ channels allow Ca^2+^ to enter the cell, which stimulates Ca^2+^ release from the SR via RyR2. Then, Ca^2+^ binds to troponin C, leading to cardiomyocyte contraction. Mitochondria are juxtaposed to the SR, and high-Ca^2+^ microdomains form at this interface. Ca^2+^-mediated crosstalk between the SR and mitochondria is mediated by RyR2 and VDAC2. Reduced mCa^2+^ uptake by MCU deletion leads to inhibition of mPTP opening. MCUb overexpression causes a reduction in mCa^2+^ uptake and consequent inhibition of mPTP opening after reperfusion. Increased NCLX activity leads to Krebs cycle impairment, bioenergetic dysfunction, a decrease in NADPH levels and ROS hyperproduction, while NCLX knockout triggers mCa^2+^ overload and consequent mPTP opening. Ca^2+^ leakage through RyR2 on the SR causes mCa^2+^ accumulation and ROS production, leading to chronic HF after mPTP opening. MFN2 ablation may reduce mPTP opening by decreasing mCa^2+^ uptake, thus protecting the heart from IRI. Deletion of both MFN1 and MFN2 in adult hearts induces lethal dilated cardiomyopathy. Abbreviations: MAMs, Mitochondria-associated membranes; SR, sarcoplasmic reticulum; RyR2, ryanodine receptor type 2; SERCA, Sarco-Endoplasmic Reticulum Calcium ATPase; MCU, mitochondrial calcium uniporter; VDAC2, voltage-dependent anion channel 2; mPTP, mitochondrial permeability transition pore; NCLX, Na^+^/Ca^2+^ exchanger; ROS, reactive oxygen species; ΔΨ_m_, mitochondrial membrane potential; MFN1, mitofusin 1; MFN2, mitofusin 2; (Created with Biorender.com).

To better understand the role of mCa^2+^ in heart physiopathology, it is necessary to know how mitochondria are organized in cardiomyocytes. The heart is a high-energy demand organ; hence, it is not surprising that mitochondria occupy 30% of the total volume of cardiomyocytes and generate approximately 95% of ATP in the body [191]. They are highly constrained among cardiac fibers and organized in three different subgroups based on their functions and location: subsarcolemmal (under the sarcolemma), perinuclear (around the nucleus) and interfibrillar (between myofibrillas) mitochondria [192]. Interfibrillar mitochondria are the most abundant, and they participate in ATP production to support myocyte contraction by regulating Ca^2+^ signaling during excitation–contraction (EC) coupling of the heart [189,193,194]. Briefly, after sarcolemma depolarization, L-type Ca^2+^ channels open to allow a small amount of Ca^2+^ to enter the cell, which stimulates even greater Ca^2+^ release from the sarcoplasmic reticulum (SR) via RyR2 (calcium-induced calcium release). Subsequently, Ca^2+^ binds to troponin C (through ATP consumption), thus inducing cardiomyocyte contraction (Figure 4). During relaxation, cytCa^2+^ is cleared by being taken back up into the SR through activation of sarco-endoplasmic reticulum calcium ATPase (SERCA) and extruded from the cell via the sarcolemma NCX [195]. Mitochondria participate in this process by regulating Ca^2+^ signaling for the production of energy required for the contraction–relaxation process [188]. However, the process through which mitochondria decode transient and rapid cytCa^2+^ signals on a beat-to-beat basis is still controversial. Over the past years, two main models have been suggested. In the first model, which was proposed by Crompton [196], mCa^2+^ influx is slow and associated with an even slower release of accumulated Ca^2+^ until a steady state is reached. Fast cytCa^2+^ oscillations are integrated by Ca^2+^ transporters in of the IMM. Therefore, changes in mCa^2+^ levels are small and associated with low energy demands by Ca^2+^ transporters. However, this slow increase in mCa^2+^ is not sufficient to stimulate ATP production at a fast enough speed to respond to the energy demand of the beating heart. Instead, the second model [197] describes quick cytCa^2+^ oscillations resulting in beat-to-beat changes in mCa^2+^ levels. Therefore, fast mCa^2+^ influx and efflux are needed. In this scenario, mCa^2+^ uptake upon each heartbeat is rapid and large enough to allow adequate energy production and to regulate cytCa^2+^ pulses. The inconsistencies in the findings related to whether beat-to-beat changes in mitochondrial calcium occur during EC coupling occur are mainly due to the methods applied to measure free mCa^2+^ levels and the species employed as experimental model (for detailed review see [193,198,199,200]).

As mentioned above, MCUC is the main route for mCa^2+^ uptake. Since this channel has a low affinity for Ca^2+^ ions, for a long time, it was questioned how mitochondria could regulate Ca^2+^ signaling during EC coupling. In this context, it is worth noting that mitochondria are juxtaposed to the SR, and high Ca^2+^-microdomains, which may help mitochondria to respond to cytCa^2+^ oscillations [201], form at the interface [202]. However, different studies using MCU-deficient models (models of global constitutive, cardiac-specific, dominant negative overexpression) [203,204,205,206] have shown no differences in basal cardiac activity, suggesting that MCU is needed only for the “fight-or-flight” response. Therefore, it is reasonable to speculate about the presence of other channels in the IMM in cardiomyocytes. Rapid uptake channels (RaMs), as their name suggests, exhibit faster Ca^2+^ influx than MCUC. However, they do not seem to be involved in beat-to-beat Ca^2+^ uptake [207]. In contrast, mitochondrial RyR (mRyR) appears to participate in mCa^2+^ uptake in the beating heart, since it allows rapid influx at low Ca^2+^ concentrations [208], although, further studies are demanded.

It is well recognized that mCa^2+^ overload induces cell death and subsequently cardiac dysfunction by activating mPTP opening [209]. mPTP is a nonspecific pore at the IMM, and its components and regulators are still under investigation (the latest findings on its structure can be found in recent reviews by Bonora et al. [210,211]. In recent decades, ATP synthase has been identified as the central core for pore formation, and our group demonstrated that disruption of ATP synthase dimers and mutation of the c-subunit, which disrupt the c-ring conformation, mediates mPTP opening [9]. Furthermore, it was recently discovered that the Ca^2+^ binding site on the catalytic portion of the ATP synthase β subunit generates conformational changes, which spread through oligomycin sensitivity conferring protein (OSCP) to the lateral stalk, ultimately inducing mPTP opening [212]. mPTP allows the free passage of molecules and ions (<1.5 kDa) and consequent ΔΨ_m_ dissipation, ROS burst and a reduction in ATP production, ultimately leading to activation of cell death pathways [213,214]. Indeed, mCa^2+^ overload and mPTP opening have been widely reported in IRI [10,215]; therefore, it was hypothesized mPTP inhibition preventing mCa^2+^ overload by deleting MCUC. Several mouse models have been proposed; however, contradictory results have been obtained. Studies on cardiac-specific knockout of MCU [204,205] confirmed the initial hypothesis; in contrast, constitutive MCU knockout [203] and cardiac-specific dominant negative overexpression of MCU [216] result in loss of mPTP opening. However, the hearts of the animal models used in these studies were not protected from IRI. These differences may suggest that deletion of MCU at an early stage can induce alternative cell death pathways. Interestingly, a recent work examined the hypothetical cardioprotective role of the MCUb subunit. In cardiomyocyte-specific MCUb-overexpressing transgenic mice, a reduction in mCa^2+^ uptake and consequent inhibition of mPTP opening after reperfusion were observed (Table 1 and Figure 4) [217].

Contractile dysfunction is also associated with a reduction in mCa^2+^ levels and consequent effects on energy supply and demand matching and results in cardiac hypertrophy and ultimately heart failure (HF). In guinea pig hearts, an increase in cytosolic Na^+^ levels due to increased activity of NCLX is associated with inhibition of the Krebs cycle, bioenergetic dysfunction, a decrease in NADPH levels and ROS hyperproduction (Figure 4) [218,219]. In contrast, studies on NCLX knockout hearts have shown that mCa^2+^ overload and consequent mPTP opening occur. (Table 1.) However, targeting of NCLX in an ischemic model to overcome Ca^2+^ accumulation in mitochondria has been proposed. Cyp D-null mice were rescued by NCLX overexpression, which reduced mPTP opening [220].

It should also be pointed out that the disruption of the contact sites between the SR and mitochondria may contribute to the dysregulation of mCa^2+^ homeostasis [221]. Ca^2+^-mediated crosstalk between these two organelles is regulated by RyR2 and VDAC2 (on SR and mitochondria, respectively) [222]. Using a murine model of post-myocardial infarction, Santulli et al. proposed the existence of a positive feedback loop between SR and mitochondria [223,224]. In this model, Ca^2+^ leakage through RyR2 on the SR causes mCa^2+^ accumulation and ROS production, which in turn leads to post-translational modification of the channel itself, causing chronic HF after mPTP opening [223,224]. Additionally, SR Ca^2+^ leakage induces the activation of spontaneous action potentials, which trigger cardiac arrhythmias [225,226].

Moreover, it has been reported that cardiac myocytes isolated from mitofusin-2 (MFN2) knockout (MFN2-KO) mice [227,228] display a reduction in mCa^2+^ uptake, resulting in inadequate ATP production. Consequently, these mice develop cardiac hypertrophy. The authors also suggested that MFN2 ablation may reduce mPTP opening by decreasing mCa^2+^ uptake, thus protecting hearts from IRI (Table 1) [227]. Furthermore, conditional gene deletion of mitofusin 1 (MFN1) and MFN2 in adult hearts induces lethal dilated cardiomyopathy [52,221].

## 4. Targeting Mitochondrial Ca^2+^ Signaling as a Promising Therapeutic Approach

It is becoming increasingly clear that targeting mCa^2+^ might be a potential and valid strategic option not only for cancer therapy but also for the treatment of neurodegenerative diseases and CVDs. In recent years, efforts have been made to decodify the mitochondrial calcium signaling network to develop selective inhibitors or regulators of calcium channels, exchangers and pumps. At the preclinical level, these strategies have demonstrated great potential, although major drawbacks have been reported when applied in vivo. Thus, approved therapies are still unavailable. This section is devoted to summarizing the most recent scientific data regarding promising therapeutic approaches targeting mCa^2+^ signaling with a particular focus on cancer, neurological disorders and CVDs.

Specifically, in the context of cancer, even though the inhibition of MCU has emerged as a promising strategy to slow tumor progression, no successful therapies have been approved to date. Interestingly, in human colon cancer and cancer-derived cells, it has been reported that the overexpression of anti-miR-25 can suppress the inhibitory effect of miR-25 on MCU expression. Thus, mCa^2^^+^ uptake is re-established, and apoptosis resistance is reversed (Table 2) [86].

Ruthenium red (RuR) and Ruthenium 360 (Ru360) are the most well-known compounds capable of inhibiting MCU activity. RuR is a nonspecific MCU inhibitor that prevents mCa^2^^+^ uptake without perturbing mitochondrial respiration or Ca^2+^ efflux [229], while Ru360 is a selective MCU inhibitor. However, because these compounds are cell impermeant, their applicability in vitro is still limited [230]. Intriguingly, the synthesis and biological activity of a novel ruthenium complex named Ru265 were recently characterized. This new ruthenium derivative is cell permeable, slightly toxic, and more strongly inhibits MCU activity than Ru360. In addition, it does not affect cytoCa^2+^ dynamics or the ΔΨ_m_. Woods and his group demonstrated that this compound is capable of protecting neonatal rat ventricular myocytes from IRI by preventing mitochondrial swelling, mPTP opening and cell death [231]. Hence, Ru265 is a novel potential drug for cardiac disorders.

DS16570511 is another recently identified effective MCU inhibitor. It shows high specificity for MCU and is cell permeant. Interestingly, it was proven to block mCa^2+^ overload in Langendorff perfused rat hearts and to increase cardiac contractility without compromising heart rate [232]. However, its ability to ameliorate AD pathologies has not yet been investigated [233]. Despite this encouraging evidence, its side effects on the ΔΨ_m_ limit its usage [234,235].

Mitoxantrone is a topoisomerase type II inhibitor that is currently used for the treatment of acute myeloid leukemia (AML) and breast cancer [230].

By using a high-throughput screening strategy, it was recently demonstrated to have a direct inhibitory effect on MCU [236]; however, it has been reported to have high cardiotoxicity [237].

Alternatively, the MAMs are another possible site of action. Indeed, it has been demonstrated that the peptide BCL-2-IP3R disruptor 2 (BIRD-2) is capable of blocking the interaction between Bcl-2 and IP3Rs, thus triggering proapoptotic Ca^2+^ signaling in cancer cells [238,239].

SERCA inhibition is another method for triggering cell death. It is widely recognized that SERCA pumps play a crucial role in cellular viability [240]. Thapsigargin selectively binds and blocks the SERCA pump. This inhibition provokes dysregulation of intracellular Ca^2+^ levels and subsequent induction of apoptotic cell death not only in cancerous cells but also in normal cells [241]. This is the main reason why the clinical application of thapsigargin has been hindered. To overcome this *impasse*, a thapsigargin prodrug called Mipsagargin G-202 was recently developed [242]. Unlike thapsigargin, G-202 does not induce systemic toxicity. In fact, it has been shown to be promising in several preclinical studies and is currently in phase II clinical trials for the treatment of prostate cancer and glioblastoma [243].

Regarding natural compounds, the polyphenol resveratrol and its derivative piceatannol display high selectiveness in increasing mCa^2+^ uptake in cancer cells after SERCA inhibition at MAMs without affecting healthy cells [244]. However, due to a low bioavailability of resveratrol, there is limited development concerning its use in clinical settings [245]. Among polyphenols, Kaempferol is a natural flavonoid emerging as a promising anti-cancer compound [246,247,248]. It has been found to be a cell permeant specific enhancer of MCU [249,250]. Interestingly, by modulating mCa^2+^ uptake, it has been recently demonstrated to activate metabolism/secretion coupling in pancreatic β-cells [251]. Moreover, in a recent study, Kaempferol showed its ability to protect cardiomyocytes from anoxia/reoxygenation (A/R) injury through reduction of ROS production, preservation of ΔΨ_m_ and inhibition of mPTP opening [252]. 

With regard to drugs used for controlling mCa^2+^ homeostasis, SB202190 is an inhibitor of p38 mitogen activated protein (MAP) kinase, which has been proven to reversibly stimulate mCa^2+^ uptake in both intact and permeabilized Hela cells [253].

Although pharmacological regulators of Ca^2+^ homeostasis, such as verapamil, a blocker of plasma membrane Ca^2+^ channels used for the treatment of arrhythmia and some form of hypertension [254], are available for the treatment of some CVDs, targeting mCa^2+^ flux remains challenging. KB-R7943, CGP-37157 and SEA0400 are NCLX inhibitors that have been demonstrated to exert promising cardioprotective effects in an animal model of HF [255,256,257]. In addition, CGP-37157 also confers neuroprotection [258,259]. However, NCLX inhibitors never entered clinical development [185,260] due to the fact that these compounds also block the plasma membrane Na^+^/Ca^2+^ antiporter SLC8A1 (also known as NCX1) [261]. Specifically, KB-R7943 has been proven to significantly protect against IR-induced damage [255,262,263] and neuronal injury [264]. In 2007, it was demonstrated that KB-R7943 is also capable of inhibiting MCU activity, leading to a reduction in mPTP opening during reperfusion, conferring a cardioprotective effect [265]. In contrast, another group confirmed that KB-R7943 inhibits Ca^2+^-induced mPTP opening but does not prevent mitochondrial calcium uptake [266]. Clearly, the mechanism through which KB-R7943 exerts protective effects remains unclear and controversial; thus, further studies are required.

Very recently, by screening a library of 44,000 compounds, Di Marco et al. discovered two MICU1 targeting compounds named MCU-i4 and MCU-i11. By directly binding to MICU1, they decrease mCa^2+^ influx both in intact cells and in muscle fibers. These novel compounds impair muscle cell growth [267], highlighting the crucial role of mCa^2+^ in muscle physiology.

Considering the crucial role of mPTP in both CVDs and neurodegenerative diseases, there is a strong interest in developing drugs to be used as therapeutic agents. However, the molecular identity of the mPTP has not yet been fully discovered, leading to difficulties in developing effective therapies.

The effects of cyclosporine A (a CypD inhibitor) have been evaluated in several clinical trials for acute myocardial infarction (AMI) [268,269]. In a pilot phase II clinical trial, it was demonstrated to decrease the infarct size [270]. In addition, in the Cyclosporine and Prognosis in Acute Myocardial Infarction Patients (CIRCUS) trial, cyclosporine A failed not only to ameliorate clinical outcomes but also to prevent adverse left ventricular remodeling at 1 year after myocardial infarction (MI) [271]. Other trials on different mPTP inhibitors and in different diseases have also failed recently [272]. At the preclinical level, studies adopting pharmacological (cyclosporine A) and genetic approaches (CypD knockout) to inhibit mPTP opening have reported reductions in neuronal injuries and degeneration in cultured cells and mutant mouse models of AD [129,273].

Interestingly, our group recently developed the first small-molecule mPTP opening inhibitors based on a 1,3,8-triazaspiro[4.5]decane scaffold, which targets the c subunit of the F_1_/F_O_-ATP synthase complex. These compounds demonstrated beneficial effects in an ex vivo model of MI without having off-target effects at the cellular and mitochondrial levels [274].

## 5. Concluding Remarks and Perspectives 

In this review, we highlighted the crucial role of mCa^2+^ in both physiological and pathophysiological conditions. Thus far, mCa^2+^ levels should be tightly regulated and balanced. In fact, as we have presented in the sections above, alterations in the amplitude or in the spatial–temporal control of mCa^2+^ signaling can provoke deleterious effects that have been linked to several pathologies, such as cancer, neurodegeneration and cardiovascular disorders.

Indeed, it is widely accepted that we are moving into an era of mitochondrial medicine, in which mCa^2+^ has gained growing attention. In recent years, strong efforts have been made in this field, leading to several opportunities to translate these findings into clinical therapies.

The identification of the key players involved in mCa^2+^ influx and efflux has led to potential therapeutic intervention such as molecules capable of efficiently and specifically inhibiting or sustaining these pathways.

To date, pharmacological strategies and methods that have been studied seem to be ineffective when tested in vivo. Since studies have shown that these strategies may have negative effects; for now, the best approach in vivo context is caution. Thus, further studies are urgently required. mCa^2+^ homeostasis modulation is at the core of the issue—drugs and therapies that target mCa^2+^ are needed.

## Figures and Tables

**Table 1:** Summary of the key regulatory proteins and transporters associated with either cancer, neurodegeneration and cardiovascular diseases.

Ca^2+^-Related Proteins and Channels	Genetic Alteration/Protein Modification	Cellular Model	Ca^2+^-Related Mechanism	References
MCU	KD	Prostate and colon cancer, TNBC, HCC	Low mCa^2+^ uptake	[86,88,89]
	Upregulation	AD	High mCa^2+^ uptake and mPTP opening	[119]
MCUb	Cardiac specific KO	Mouse cardiomyocyte	Low mCa^2+^ uptake and mPTP opening reduction	[204,205]
	OE	Mouse cardiomyocytes	Low mCa^2+^ uptake and mPTP opening inhibition	[217]
MICU1	Akt-mediated phosphorylation	Renal, ovarian, breast, and lung cancer	High mCa^2+^ uptake	[91]
NCLX	downregulation	AD mouse and human brains	High mCa^2+^ uptake	[130]
	Tamoxifen-induced deletion	Adult mouse hearts	High mCa^2+^ uptake and mPTP opening	[220]
MFN2	KO	Mouse cardiac myocytes	Low mCa^2+^ uptake and mPTP opening reduction	[227]
BAP1	IP3R3	Mesothelioma	Low ER- Ca^2+^ release	[98]
	deubiquitylation			
	and stabilization			
PTEN	downregulation	Prostate and lung cancer	Low ER- Ca^2+^ release	[97]
PS1	Mutation	Animal model of AD	High ER- Ca^2+^ release	[117,118]
PS1/PS2	PS1 (mutation M146L) and PS2 (mutation N141I)	FAD	High ER- Ca^2+^ release	[116]
LRKK2	mutation	Fibroblasts from PD patients	High mCa^2+^ uptake	[182]
PINK1	Mutation	Dopaminergic neurons	High mCa^2+^ levels and increased ER–mitochondria contact sites	[174]
Parkin	Deficiency or mutation	PD patient fibroblasts	Low mCa^2+^ uptake and reduced ER–mitochondria contact sites	[180]

Abbreviations: KD, knockdown; OE, overexpression; KO, knock-out; AD, Alzheimer disease; FAD, familial Alzheimer disease; PD, Parkinson disease; TNBC, triple-negative breast cancer; HCC, hepatocellular carcinoma.

**Table 2:** List of modulators available for shaping mCa^2+^ signaling as potential therapeutic approach.

Therapeutic Target	Compound	Side Effects	Cell Permeability	In Vivo Applicability	References
**MCU**	anti-miR25	Not observed	Yes	ND	[86]
	Ru265	Not observed	Yes	ND	[231]
	DS16570511	ΔΨ_m_ loss and cell death	Yes	No	[232,234,235]
	Mitoxantrone	Cardiotoxicity	Yes	Limited	[236,237]
	Kaempferol	Not observed	Yes	Yes	[249,250]
	SB202190	Not observed	Yes	ND	[249]
	KB-R7943	Not selective	Yes	ND	[265]
**MICU1**	MCU-i4 and MCU-i11	Not observed	Yes	ND	[267]
**NCLX**	KB-R7943,	Not selective	Yes	ND	[255,262,264]
	CGP-37157	Not observed	Yes	ND	[257,259]
	SEA0400	Not observed	Yes	ND	[255,256]
**mPTP**	Cyclosporin A	Not observed	Yes	ND	[270]
	1,3,8-Triazaspiro[4.5]decane derivatives	Not observed	Yes	ND	[274]
**SERCA**	Thapsigargin	Not selective	Yes	No	[241]
	Mipsagargin G-202	Not observed	Yes	Yes	[243]
	Resveratrol and piceatannol	Not observed	Yes	Limited bioavailability	[244]
**MAMs: BCl2-IP3R3**	BIRD-2	Not observed	Yes	ND	[238,239]

Abbreviations: ND, not determined.

## Abbreviations

AD	Alzheimer disease
APP	Amyloid precursor protein
Aβ	Amyloid β-peptide
APOE	Apolipoprotein E
BAP1	BRCA1-associated protein-1
CaMKI	Ca^2+^/calmodulin-dependent protein kinase
Ca^2+^	Calcium
CaM	Calmodulin
CVDs	Cardiovascular diseases
CDKs	Cyclin-dependent kinases
CypD	Cyclophilin D
cytCa^2+^	Cytosolic calcium
ETC	Electron transport chain
ER	Endoplasmic reticulum
EMRE	Essential MCU regulator
FAD	Familial AD
HCX	H^+^/Ca^2+^ exchangers
HF	Heart failure
HD	Huntington disease
IMM	Inner mitochondrial membrane
IP3R3	Inositol 1,4,5-trisphosphate (IP3) receptor type 3
IP3Rs	Inositol 1,4,5-trisphosphate (IP3) receptors
IMS	Intermembrane mitochondrial space
IRI	Ischemia/reperfusion injury
IDH	Isocitrate dehydrogenase
KGDH	Ketoglutarate dehydrogenase
LRRK2	Leucine-rich repeat kinase 2
MAMs	Mitochondrial associated membranes
MCUC	Mitochondrial calcium Uniporter Complex
MCU	Mitochondrial calcium Uniporter
MICU1	Mitochondrial calcium uptake 1
mCa^2+^	Mitochondrial calcium
ΔΨm	Mitochondrial membrane potential
mPTP	Mitochondrial permeability transition pore
mROS	Mitochondrial ROS
MFN2	Mitofusin 2
MI	Myocardial infarction
NCLX	Na^+^/Ca^2+^ exchangers
OMM	Outer mitochondrial membrane
OXPHOS	Oxidative phosphorylation
PD	Parkinson disease
PTEN	Phosphatase and tensin homolog
PS1	Presenilin-1
PS2	Presenilin-2
PINK1	PTEN-induced kinase 1
PDH	Pyruvate dehydrogenase
ROS	Reactive oxygen species
Ru360	Ruthenium 360
RuR	Ruthenium red
RyR2	Ryanodine receptor (RyR) type 2
RyR	Ryanodine receptor
SERCA	Sarco-Endoplasmic Reticulum Calcium ATPase
SR	Sarcoplasmic reticulum
SAD	Sporadic AD
SOCE	Store-operated calcium entry
TRP	Transient receptor potential
VDAC	Voltage dependent anion channel
VGCC	Voltage-gated calcium channel
